# Targeted inhibition of ferroptosis in bone marrow mesenchymal stem cells by engineered exosomes alleviates bone loss in smoking-related osteoporosis

**DOI:** 10.1016/j.mtbio.2025.101501

**Published:** 2025-01-21

**Authors:** Yao Wang, Lin Sun, Zhenglin Dong, Tianyu Zhang, Leining Wang, Yihui Cao, Hui Xu, Chenglei Liu, Bo Chen

**Affiliations:** a Department of Hand and Foot Surgery, Beilun Branch of the First Affliated Hospital, College of Medicine. Zhejiang University, Ningbo, China; b Department of Orthopedic Surgery, The First Affliated Hospital, Zhejiang University School of Medicine, Hangzhou, China; c Department of Radiology, Shanghai Ninth People's Hospital, Shanghai Jiao Tong University School of Medicine, Shanghai, China; d Department of Orthopedic Surgery, Shanghai Ninth People's Hospital, Shanghai Jiao Tong University School of Medicine, Shanghai, China; e Bengbu First People's Hospital, Bengbu, China

## Abstract

Smoking-related osteoporosis (SROP) is characterized by reduced bone mass, primarily due to the accumulation of tobacco-derived toxins. This study demonstrates the activation of ferroptosis and reactive oxygen species (ROS)-related pathways in the bone marrow mesenchymal stem cells (BMSCs) of SROP mice. Here, we integrated genetic engineering and bone-targeting peptide modification to develop innovative bone-targeting engineered exosomes. Using genetic engineering techniques, we introduced α-1,3-fucosyltransferase 6 (Fut6), a key protein involved in prostate cancer bone metastasis, and identified exosomes expressing Fut6 (F6-exo) with bone-targeting capabilities. Additionally, we modified these exosomes with a bone-targeting peptide, (AspSerSer)6, to synthesize F6-(DSS)_6_-exo. F6-(DSS)6-exo enabled the targeted delivery of curcumin, restoring the osteogenic differentiation potential of BMSCs and mitigating bone loss in SROP mouse models. In summary, this study highlights the combination of genetic engineering and hydrophobic diacylglycerol insertion as a novel targeted therapeutic approach for SROP.

## Graphical abstract

**Figure:**
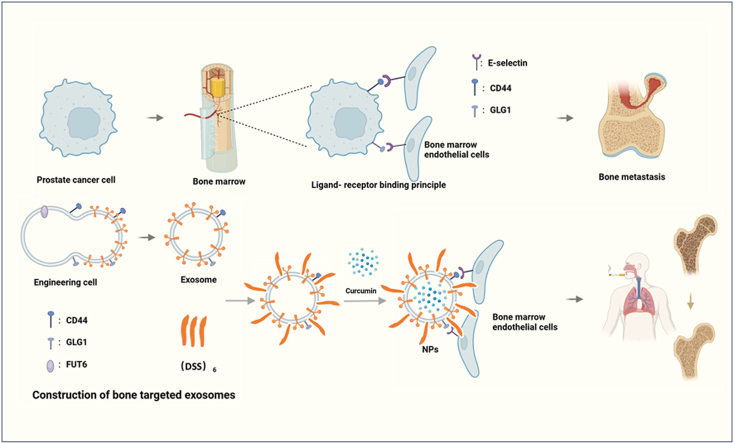

## 1 Introduction

Smoking, recognized as one of the most detrimental behaviors, is a modifiable risk factor for preventing premature death [1]. Upon inhalation, harmful substances such as nicotine and tar rapidly enter the bloodstream through the lungs and accumulate within the body [2]. These toxins exert chronic adverse effects on multiple organ systems, including the respiratory, circulatory, and digestive systems [3,4]. Additionally, once tobacco-derived toxins reach the skeletal system via the bloodstream, they trigger inflammation, impair bone nutrition, and disrupt bone metabolism [[5], [6], [7]]. These processes can lead to conditions such as arthritis, infections, non-healing fractures, and osteoporosis [[8], [9], [10], [11]]. Notably, smoking has been identified as an independent risk factor for osteoporosis progression and a direct cause of SROP [12,13]. Bone remodeling depends on a delicate balance between osteoblast-mediated bone formation and osteoclast-mediated bone resorption [14]. Tobacco toxins disrupt this balance, thereby contributing to the development of osteoporosis.

Research has demonstrated that cigarette toxins impair bone formation [15]. Osteoblastic bone formation is mediated by bone marrow mesenchymal stem cells (BMSCs), which serve as progenitors for osteoblasts and adipocytes [16,17]. BMSCs are anchored to the endothelial lining of blood vessels, creating the BMSC microenvironment [18]. In SROP, toxins such as nicotine induce oxidative stress in BMSCs, promote ferroptosis, and reduce their osteogenic differentiation potential [15,19]. Therefore, targeting BMSCs represents a promising therapeutic strategy to restore osteogenic potential and enhance osteoblastogenesis in SROP.

Bone-targeting peptides, particularly aspartic acid-serine-serine (Asp-Ser-Ser), exhibit a high affinity for calcium phosphate compounds [20]. Among these, the six-repeat sequence Asp-Ser-Ser, referred to as (DSS)_6_, has demonstrated the strongest affinity for calcium phosphate. Studies suggest that incorporating (DSS)_6_ into exosomes through hydrophobic interactions can generate exosomes with bone-targeting delivery capabilities [21]. However, the number of peptide segments that can be integrated into the exosomes is limited after co-incubation with bone-targeting peptides, and these segments are susceptible to elution [22]. As a result, the delivery efficiency of exosomes modified with bone-targeting peptides remains constrained.

Bone marrow endothelial cells constitutively express E-selectin, which facilitates the homing of hematopoietic stem cells expressing E-selectin ligand (ESL) to the bone marrow [23]. Interestingly, human prostate cancer cells also express similar ESLs, enabling their interaction with and colonization in the bone marrow [24]. Recent research has identified ESLs expressed by prostate cancer cells, such as CD44 and Golgi glycoprotein 1 (GLG1) [25,26]. Exosomes, natural extracellular vesicles, exhibit excellent biocompatibility and stability [27,28]. Building upon research into bone metastasis in prostate cancer, we hypothesize that incorporating prostate cancer bone metastasis-related proteins into exosomes can facilitate bone-targeted therapy via ligand-receptor interactions. However, ESL has a wide variety of types and complex functions [25]. The binding effect of a single ESL with E-selectin is unclear. Therefore, introducing ESL upstream molecules is a more efficient way of construction. ESLs attach to the extracellular domain of glycoproteins or glycolipids. The synthesis of ESLs on such glycoproteins is catalysed in the Golgi compartment by members of the glycosyltransferase gene family. The final step involves the transfer of fucose to N-acetylglucosamine at the terminal a-2, 3 sialo-lactosamine unit by a-1, 3-fucosyltransferases (Futs) [29]. Futs play an important role in cancer bone metastasis by catalyzing ESL. Studies have demonstrated that the overexpression of α-1,3-fucosyltransferase 6 (Fut6) in mouse cancer cells promotes the production of abundant ESLs and induces aggressive bone marrow metastasis [25,29].

In this study, we prepared Fut6-overexpressing engineered HEK293T cells. Extracellular vesicles derived from these engineered cells exhibited bone marrow-targeting capabilities (Fig. 4G). Subsequently, the bone-targeting peptide (DSS)_6_ was inserted into F6-exo to create F6-(DSS)_6_-exo. Compared to exosomes modified with (DSS)_6_ alone, F6-(DSS)_6_-exo demonstrated significantly enhanced bone-targeting efficacy. Transcriptome sequencing revealed upregulation of ferroptosis and Reactive Oxygen Species (ROS) -related signaling pathways in the BMSCs of SROP mice. Ferroptosis and ROS are key mechanisms impairing the osteogenic differentiation potential of BMSCs [30]. During intracellular divalent iron ion overload, hydroxyl radicals are generated in large quantities via the Fenton reaction, and excessive ROS induces oxidative stress, further impairing the osteogenic differentiation of BMSCs [31]. Curcumin, a polyphenolic compound extracted from turmeric, is recognized for its free radical scavenging, antioxidant, and anti-inflammatory properties [[32], [33], [34]]. Additionally, curcumin functions as an iron chelator and is one of the most widely studied ferroptosis inhibitors [31,35]. Thus, combining engineered exosomes with curcumin to create F6-(DSS)_6_-exo@Cur offers potential for treating SROP in mice by reducing ROS and inhibiting ferroptosis. Our results demonstrated that F6-(DSS)_6_-exo@Cur restored the osteogenic differentiation potential of BMSCs in SROP and reduced bone loss in mice.

## 2 Material and methods

### 2.1 Materials

Cigarettes were purchased from the local market. Curcumin was purchased from Shanghai Yuanye Bio-Technology Co., Ltd. (Shanghai, China). Penicillin-streptomycin, MEM-α medium, phosphate-buffered saline (PBS), and fetal bovine serum (FBS) were purchased from Thermo Fisher Scientific (USA). Commercial detection kits for ROS and JC-1 were purchased from Beyotime (Shanghai, China). The ferrous ion detection kit was supplied by Elabscience Co., Ltd. (China). Alkaline phosphatase and Alizarin Red staining solutions were obtained from Beyotime (Shanghai, China) and Cyagen (China), respectively. Antibodies used included Anti-GPX4 (Cat#: ab125066; Abcam), Anti-CD44 (Cat#: ab254530; Abcam), Anti-GLG1 (Cat#: ab271182; Abcam), Anti-Alix (Cat#: ab275377; Abcam), Anti-Hsp70 (Cat#: ab2787; Abcam), Anti-TSG101 (Cat#: ab133586; Abcam), and Anti-GAPDH (Cat#: 60004-1-Ig; Proteintech) at a dilution of 1:50,000. Retroviral vectors and two helper plasmids were designed by OBiO Technology (Shanghai, China).

### 2.2 Cigarette smoke extract (CSE) preparation

Cigarette smoke was aspirated using a negative-pressure pump and bubbled into sterile PBS in 15 mL BD Falcon tubes. Each smoking cycle involved a 2-s smoking duration followed by a 60-s interval. A total of 10 mL of CSE was prepared from every 5 cigarettes. The CSE solution was filtered through a 0.22 μm-pore filter. The concentration of CSE was determined spectrophotometrically at 320 nm.

### 2.3 Cell culture

BMSCs were isolated from the bone marrow of male C57BL/6J mice. Cells were cultured in α-MEM medium (Gibco, USA) supplemented with 10 % fetal bovine serum (Gibco, USA) and 100 μg/mL penicillin-streptomycin. Cultures were maintained at 37 °C in a humidified atmosphere containing 5 % CO₂.

### 2.4 Animals

All animal experimental protocols were approved by the Animal Ethics Committee of the Shanghai Ninth People's Hospital, Shanghai JiaoTong University School of Medicine. Male C57BL/6 mice were supplied by Shanghai Jihui Laboratory Animal Care Co., Ltd. (Shanghai, China). To establish the mouse SROP model, animals were intraperitoneally injected with 100 μL of PBS or CSE every other day between 2:00 and 4:00 p.m. as described in the literature [36]. After 4 weeks, the mice were euthanized via CO₂ asphyxiation.

### 2.5 Retroviral production and transduction

Retroviral vectors encoding human Fut6 and retrovirus packaging helper plasmids were designed and synthesized by OBIO Biotechnology Co., Ltd. (Shanghai, China). Retroviral vectors and two helper plasmids were transfected into HEK293T cells using TransIT-LT1 Transfection Reagent (Mirus Biology, Madison, WI, USA). Viral supernatants were collected 48 h post-transfection. HEK293T cells were incubated with the viral supernatants in the presence of 8 μg/mL polybrene (Santa Cruz Biotechnology, USA) for 24 h to achieve transduction.

### 2.6 Isolation and characterization of exosomes

Cell lines were cultured until 70 % confluence in standard medium, after which the medium was replaced with 10 % exosome-depleted FBS. Cells were then maintained under normal conditions for 48 h. The supernatant was collected and subjected to sequential centrifugation steps (300×*g* for 10 min, 2,000×*g* for 10 min, 10,000×*g* for 30 min, and 110,000×*g* for 90 min). The resulting pellet was resuspended in PBS, and exosomes were isolated using ExoQuick Reagent (System Biosciences, USA). The protein concentration of the exosomes was determined using a bicinchoninic acid (BCA) protein assay kit (Beyotime, Shanghai, China). The size distribution of exosomes was analyzed through electrophoresis and Brownian motion video analysis using laser scattering microscopy. Transmission electron microscopy (TEM) was employed to capture microscopic images of exosomes. Western blot analysis was conducted to confirm the presence of exosome-specific markers, including Alix, Hsp70, and TSG101.

### 2.7 Insertion of bone-targeting peptides and drug loading

To synthesize DSPE-PEG-Mal-(DSS)_6_, DSPE-PEG-Mal and (DSS)_6_ were reacted in a 1:1 M ratio in 5 mL DMF(N,N-dimethylformamide) at room temperature for 24 h via maleimide coupling to synthesize DSPE-PEG-Mal-(DSS)_6_. To facilitate the conjugation of Exo with the bone-targeting peptide, 10 μL Exo (10^12^ particles/mL) was gently mixed with 90 μL DSPE-PEG-Mal-(DSS)_6_ (10 μM) in 100 μL PBS, followed by overnight incubation at 4 °C. To remove unbound DSPE-PEG-Mal-(DSS)_6_, the mixture was centrifuged at 10^5^×g for 70 min at 4 °C, washed with PBS, and then resuspended in PBS. For curcumin loading, given its hydrophobic nature, curcumin can passively diffuse into the exosome interior across the membrane concentration gradient, though with relatively low efficiency. We employed ultrasound treatment to reduce the rigidity of the exosomal membrane, thereby enhancing its permeability and allowing more curcumin to diffuse into the exosomes. After mixing (DSS)6-exo with curcumin in PBS, the mixture was subjected to ultrasound treatment at room temperature for 1 min (100 W, 5 s sonication, intermittent 5 s) to facilitate loading. The mixture was then statically incubated for 30 min to restore membrane integrity. Since the free DSPE-PEG-Mar-(DSS)6 had already been eluted, the peptides conjugated to the lipid membrane did not enter the exosomes.

### 2.8 Micro-CT analysis

The right tibia of each mouse was scanned using micro-computed tomography (Micro-CT) to analyze the microstructure of trabecular and cortical bone beneath the tibial plateau growth plate. Scanning parameters included a current of 114 μA, an integration time of 300 ms, a resolution of 36 μm, and a voltage of 70 kV. The region of interest was located 1.9 mm distal to the proximal tibial condyle, adjacent to the growth plate. The bone microstructures of the tibia were analyzed individually using Scanco Medical AG IPL software (version 4.29d, Switzerland). Parameters of interest included trabecular thickness (Tb.Th, mm), trabecular number (Tb.N, 1/mm), trabecular separation (Tb.Sp, mm), and bone volume fraction (BV/TV, %).

### 2.9 Intracellular ROS assay

After different treatments, cells were washed three times with PBS and then treated with ROS detection kit (Beyotime, Shanghai, China). After incubation at 37 °C incubator for 20 min, the cells were washed three times with PBS. The results were observed by fluorescence microscope (Olympus).

### 2.10 Ferrous ion detection

After different treatments, cells were rinsed three times with PBS and then treated with ferrous ion detection kit (Elabscience, China). After incubated at 37 °C incubator for 1 h, the cells were washed three times with PBS. The results were observed by fluorescence microscope (Olympus).

### 2.11 JC-1 staining

Aspirate the culture solution, wash the cells once with PBS or other appropriate solution, and add 1 mL of cell culture solution. Add 1 mL of JC-1 staining solution and mix well. Incubate for 20 min at 37 °C in a cell culture incubator. At the end of incubation, aspirate the supernatant and wash twice with JC-1 staining buffer. Add 2 mL of cell culture solution. Observe under laser confocal microscope (Olympus).

### 2.12 MDA assay

Cells were seeded in a 6-well cell culture plate and homogenized. The protein concentration was determined using a BCA Protein Assay Kit (P0010S, Beyotime, China). MDA levels were measured using a Lipid Peroxidation MDA Assay Kit (M496, Dojindo Laboratories, China). The ratio of MDA levels to protein concentration was calculated.

### 2.13 Exosome uptake assay

The exosome suspension was diluted to an appropriate concentration (10^11^ particles/mL). DiO dye (2 μM) was added to the exosome solution and gently mixed. The mixture was incubated at room temperature in the dark for 30–60 min, with gentle mixing every 10–15 min. Unbound dye was removed using ultracentrifugation or filtration, yielding DiO-labeled exosomes. Target cells (e.g., BMECs or fibroblasts) were seeded onto a 6-well plate and cultured to the appropriate density (typically 60%–80 % confluence). DiO-labeled exosomes were collected and added to the cell culture medium. The cells were incubated with exosomes at 37 °C and 5 % CO₂. After incubation, the cells were gently washed with PBS to remove uninternalized exosomes. The cells were then fixed with 4 % paraformaldehyde for 15–30 min and observed using a fluorescence microscope.

### 2.14 Immunohistochemical staining

In short, using ethanol solution for stepwise dehydration. Repair the slices using a repair kit containing sodium citrate buffer at 95 °C/10 min. Choose to drop 3 % hydrogen peroxide solution onto tissue slices and incubate in a dark room for 30 min. Incubate the slices with 0.3 % TritonX-100 at room temperature. Add 3 % BSA dropwise onto the immunohistochemistry area and seal at room temperature for 30 min. Then wash with PBS. Add diluted primary antibody dropwise to the immunohistochemistry area and incubate overnight at 4 °C, and the corresponding secondary antibody (Cat#:ab6721; Abcam) for 1 h at room temperature.

### 2.15 Alkaline phosphatase staining

Seven days after the cells were induced with osteogenic induction medium, the cells were washed three times with PBS, fixed with 4 % paraformaldehyde for 30 min at room temperature, and washed three times with PBS. The cells were stained with alkaline phosphatase staining solution (Beyotime, China) for 1 h at room temperature and then the staining solution was aspirated. The staining solution was washed twice with PBS and observed under a microscope.

### 2.16 Alizarin red staining

Cells were induced using osteogenic induction medium (oricell, China). The osteogenic medium was changed every 2–3 days and the cells were cultured at 37 °C with 5 % CO2 for about 21 days. The induction was terminated according to the precipitation of calcium salt crystals and the formation of calcium nodules. Aspirate the induction medium, rinse the cells twice with PBS and fix them in 4 % paraformaldehyde for 15 min at room temperature. The fixative was aspirated, rinsed twice with PBS, and stained with alizarin red staining solution (oricell, China) for 1 h at room temperature. The staining solution was aspirated, rinsed twice with PBS and observed under the microscope.

### 2.17 RNA extraction and RT-qPCR

For the extraction of total RNA, cells were treated with the AxyProp multi-source RNA Miniprep kit (catalog number: AP-MNMS-RNA-250, Axygen, Corning, New York, USA). For reverse transcription, TaKaRa reverse transcription reagent (catalog number: D2680A, TaKaRa, Japan) was used to synthesize cDNA. Perform RT qPCR using QuantStudio 6 Flex RTqPCR system (Applied Biosystems, CA, USA) and SYBR Green PCR Mix (Cat #: B21402, Bimake, TX, USA). Calculating the relative RNA level using the comparative threshold cycle (2-ΔΔCT) method and normalizing it to the positive control value in the sample.

### 2.18 Western blot analysis

For total protein extraction, cells were lysed using cell lysis buffer (Beyotime, Shanghai, China), supplemented with a protease inhibitor (Abmole Bioscience, TX, USA), for 15 min, followed by sonication. The collected protein solution was combined with Sampling Buffer (Beyotime) and incubated at 99 °C for 10 min. Subsequently, proteins were separated using 4–20 % ExpressPlus™ PAGE Gel (GenScript, Nanjing, China) and electrophoresed in Tris-MOPS-SDS Running Buffer (GenScript), diluted with ddH2O. The gels were then electroblotted onto 0.22-μm PVDF membranes (MilliporeSigma, Massachusetts, USA). To block the membranes, 5 % BSA-TBST (Tris-buffered saline (TBS)-0.1 % Tween 20) (Beyotime) was applied for 1 h at room temperature. Incubate PVDF membranes with antibodies against GPX4 (Cat#:ab125066; Abcam), CD44 (Cat#:ab254530; Abcam), GLG1 (Cat#: ab271182; Abcam), Alix (Cat#:ab275377; Abcam), Hsp70 (Cat#:ab2787; Abcam), TSG101 (Cat#: ab133586; Abcam), and GAPDH (Cat#: 60004-1-Ig, Proteintech, 1:50000). in 4 °C. The next day, the membranes were washed with PBS and then incubated with an appropriate secondary antibody (Cat#: SA00001-1 and SA00001-2, Proteintech, 1:5000) in 2 % non-fat milk for 1 h. The immunoreactive bands were detected using a chemiluminescence kit (Cat#: RPN2232, Amersham Biosciences Ltd., UK) and then analyzed using Image-Pro Plus software (version 6.0).

### 2.19 Statistical analysis

Data are presented as mean ± standard deviation (SD) and results are presented as bar graphs. Statistical analysis was performed using Prism Version 9 software (GraphPad, California, USA). Unpaired two-tailed Student's t-test was used for pairwise comparisons between two groups. Comparisons involving three or more groups were analyzed using one-way analysis of variance (ANOVA) followed by Tukey's post hoc test.

## 3 Results

### 3.1 CSE exposure resulted in bone loss in mice

**Fig. .1:**
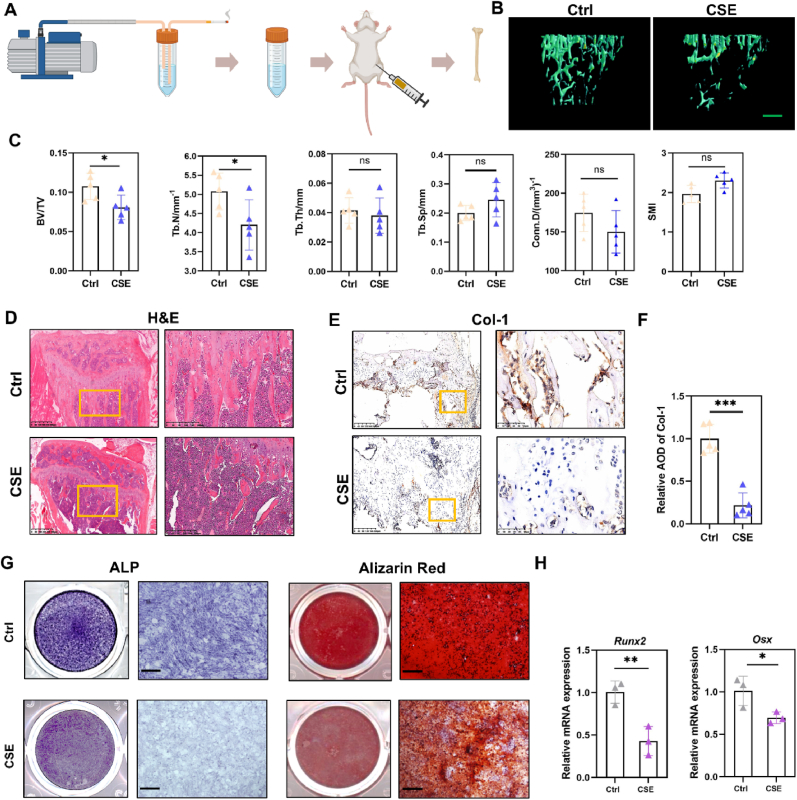
CSE exposure resulted in bone loss in mice. (A) Preparation method of CSE and SROP models. (B) Microscopic CT three-dimensional reconstruction of proximal tibia in control group and CSE group mice. Scale bar: 200 μm. (C) Quantitative statistics of bone trabecular parameters (BV/TV, Tb. N, Tb. Sp, Tb Th,Conn.D and SMI). (n = 5 in each group). (D) HE staining of proximal tibia. Scale bar: 625 μm and 300 μm. (E) Col-1 IHC staining of distal femur in control group and CSE exposed group. Scale bar: 200 μm and 50 μm. (F) The calculation of relative average optical density (AOD) of Col-1 (n = 5 in each group). (G) ALP and Alizarin Red staining. Scale bar: 250 μm. (H) qPCR analysis of osteogenesis-related genes (Runx2, Osx) of BMSCs originated from mice in the Control group and the CSE-exposed group (n = 3 in each group). ∗P < 0.05, ∗∗P < 0.01,∗∗∗P < 0.001. Student's *t*-test.

To establish a mouse model of SROP, cigarette smoke extract (CSE) was prepared and administered via intraperitoneal injection for four weeks, as shown in Fig. 1A. Three-dimensional micro-CT reconstruction of the tibia revealed a significant reduction in bone density in CSE-treated mice (Fig. 1B). Compared with the control group, the bone volume-to-total volume (BV/TV) ratio in the CSE group decreased by 24.83 %, and the trabecular number (Tb.N) was reduced by 17.17 % (Fig. 1C). Hematoxylin-eosin (H&E) staining further demonstrated a noticeable reduction in trabecular bone within the bone marrow cavity of the CSE group (Fig. 1D). Additionally, the number of osteoblasts expressing collagen type I alpha 1 chain (Col-1) was significantly lower in the CSE group compared to the control group (Fig. 1E and F). These findings indicate that CSE exposure induces osteoporosis in mice, primarily through an imbalance between bone formation, mediated by osteoblasts, and bone resorption, mediated by osteoclasts. Since osteoblasts differentiate from primitive BMSCs, we next evaluated the osteogenic differentiation potential of BMSCs in CSE-exposed mice. Following osteogenic induction, alkaline phosphatase staining and Alizarin Red staining revealed a marked reduction in the osteogenic differentiation capacity of BMSCs in the CSE group (Fig. 1G). Consistent with these staining results, the mRNA levels of osteogenic markers, including osterix (Osx) and runt-related transcription factor 2 (Runx2), were significantly decreased (Fig. 1H).

### 3.2 Transcriptome analysis and phenotype validation indicate that CSE induces ferroptosis in BMSCs

**Fig. 2:**
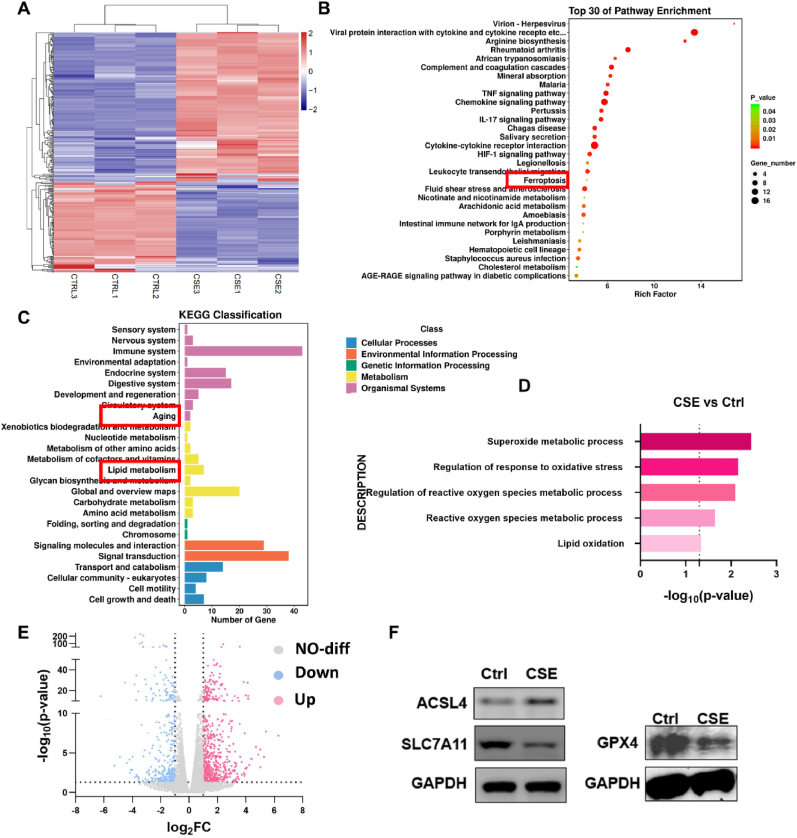
CSE exposure activates ferroptosis signaling in mice BMSCs.(A) Heatmap showed differentially expressed genes (DEGs) between BMSCs from the Control and the CSE group (n = 3 in each group). Red: up-regulated expression levels. Blue: down-regulated expression levels. (B) KEGG analysis displays the top 30 signaling pathways. (C) The KEGG classification of differentially expressed genes in B class. (D) GO analysis of ROS and lipid peroxidation related signaling pathways. (E) The volcano plot shows differentially expressed genes (DEGs) between the Control and the CSE group. (F) Western blotting (WB) analysis of ACSL4, Slc7a11 and GPX4 in two groups.

To investigate the mechanisms underlying the impaired osteogenic differentiation of BMSCs following CSE exposure, we isolated BMSCs from control and CSE-treated mice and performed transcriptome sequencing. A heatmap of differentially expressed genes revealed significant alterations in gene expression profiles in the CSE group compared to controls (Fig. 2A). Kyoto Encyclopedia of Genes and Genomes (KEGG) pathway analysis identified the top 30 signaling pathways (Fig. 2B), highlighting significant activation of the “Ferroptosis” pathway. Additionally, KEGG classification of differentially expressed genes within the B class emphasized the relevance of pathways related to “lipid metabolism” and “aging” (Fig. 2C). These findings suggest that CSE exposure leads to lipid peroxidation and ferroptosis in BMSCs, contributing to cellular aging. Gene Ontology analysis further demonstrated significant upregulation of pathways associated with ROS production and lipid peroxidation in the CSE group (Fig. 2D). Given the central role of ROS and lipid peroxidation in ferroptosis, these bioinformatics findings strongly implicate ferroptosis as the primary driver of impaired osteogenic differentiation in CSE-exposed BMSCs.The volcano plot shows that a large number of genes, including key molecules involved in ferroptosis, are altered in CSE-exposed BMSCs (Fig. 2E). To validate the reliability of the KEGG and GO analyses, we assessed key ferroptosis-related molecules (ACSL4, SLC7A11, and GPX4) [[37], [38], [39], [40]]. The results showed that the CSE group upregulated the ferroptosis-promoting molecule ACSL4, while downregulating the ferroptosis-inhibiting molecules SLC7A11 and GPX4 (Fig. 2F).

**Fig. 3:**
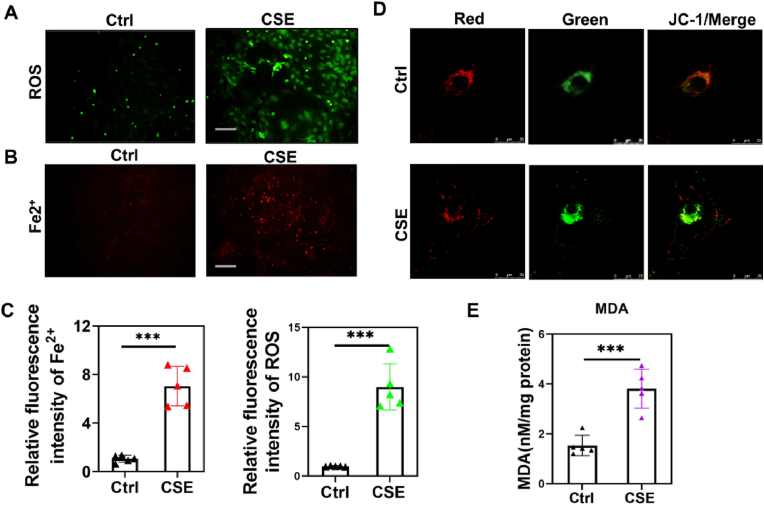
Intracellular ROS and ferrous ions were upregulated in BMSCs of CSE group. (A) Probe detection of intracellular ROS in two groups of cells. ∗∗∗P < 0.001. (B) Probe detection of intracellular ferrous ions in two groups of cells. ∗∗∗P < 0.001. (C) Relative fluorescence quantification of ROS and ferrous ions. (D) JC-1 staining of two groups of cells. (E) MDA content measurement in two groups of cells. ∗∗∗P < 0.001. Student's *t*-test.

We further evaluated ferroptosis markers in BMSCs from both groups. CSE exposure resulted in a marked increase in intracellular ROS levels (Fig. 3A–C) and an overload of ferrous ions (Fig. 3B and C). JC-1 staining demonstrated a significant decrease in the red/green fluorescence ratio in the CSE group, indicating reduced mitochondrial membrane potential (Fig. 3D). Similarly, the lipid peroxidation product Malondialdehyde (MDA) was significantly upregulated in the BMSCs of the CSE group (Fig. 3E). Collectively, these findings demonstrate that cigarette toxins elevate intracellular ROS levels and trigger ferroptosis in BMSCs. To verify whether ferroptosis is the primary mechanism underlying the impaired osteogenic differentiation of CSE-exposed BMSCs, we added ferroptosis inhibitors to the culture medium of BMSCs.The results indicate that the addition of ferroptosis inhibitors can rescue osteogenic differentiation in CSE-exposed BMSCs (Figs. S1A–B).

**Fig. .4:**
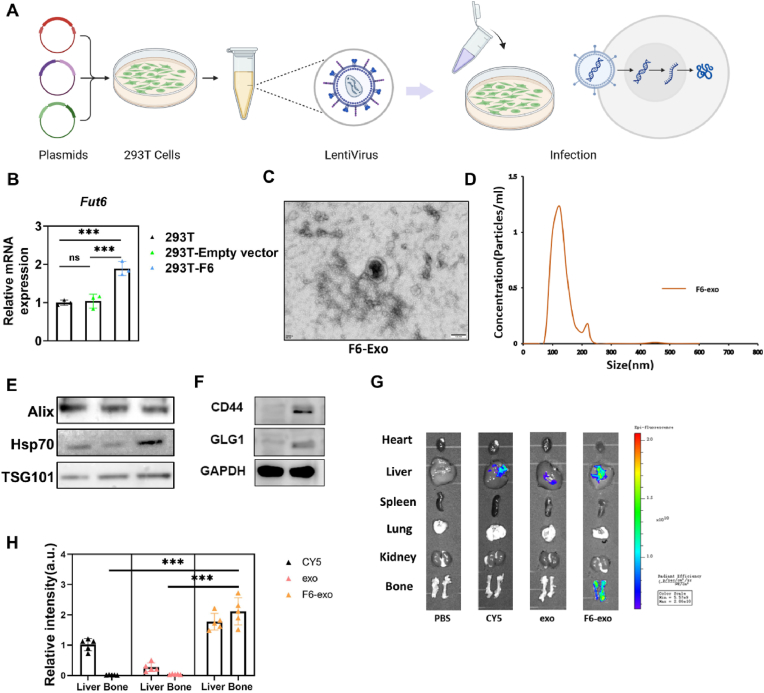
Construction of bone targeted delivery system. (A) Preparation of Fut6 overexpression cell lines, including packaging and infection of lentiviruses. (B) qPCR analysis of Fut6 in 293T cells. (C) Morphological image of F6-exo revealed by transmission electron microscope. (D) Diameter distribution of F6-exo revealed by nanoparticle tracking analysis (NTA). (E) The protein level of Alix, HSP70 and TSG101 of F6-exo. (F) Western blot analysis of CD44 and GLG1 in exosomes drived from 293T cells. (G) Biophotonic imaging analysis revealed the organ distribution of Cy5-labeled F6-exo 4 h after intravenous tail vein injection.(H) Relative fluorescence quantification of Cy5. ∗∗∗P < 0.001. One-way ANOVA.

### 3.3 Construction of engineered exosomes through genetic engineering

Inspired by studies on prostate cancer bone metastasis, we selected Fut6 as the engineering gene [2]. To construct engineered exosomes, 293T cells were transfected with a retroviral vector encoding Fut6 along with retroviral packaging helper plasmids to produce lentiviral particles. These lentiviral particles were subsequently used to infect 293T cells, generating a cell line that overexpresses Fut6 (Fig. 4A). mRNA detection confirmed the successful genetic modification of 293T cells (Fig. 4B). Exosomes were then isolated from the engineered 293T cells and designated as F6-exo. TEM and nanoparticle tracking analysis (NTA) revealed that F6-exo conformed to the typical morphological characteristics of exosomes (Fig. 4C and D). Western blot analysis further confirmed the presence of extracellular vesicle markers, including HSP70, TSG101, and Alix, in F6-exo (Fig. 4E). As described previously, CD44 and GLG1 are both substrates of Fut6 and ligands for the E-selectin receptor on the surface of bone marrow endothelial cells [25,26]. Compared to the control group, Fut6 overexpression significantly increased CD44 and GLG1 levels in F6-exo (Fig. 4F). To evaluate whether F6-exo could specifically target bone tissue in vivo, Cy5-labeled F6-exo was intravenously injected into mice, followed by biophotonic imaging analysis. Results demonstrated that F6-exo selectively accumulated in bone tissue (Fig. 4G and H). Similarly, the introduction of Fut6 increased the uptake of exosomes by bone marrow endothelial cells, a phenomenon not observed in cells from other tissues (Figs. S2A–B).

### 3.4 Preparation of bone-targeted nanoparticles and safety assessment *in vivo*

**Fig. 5:**
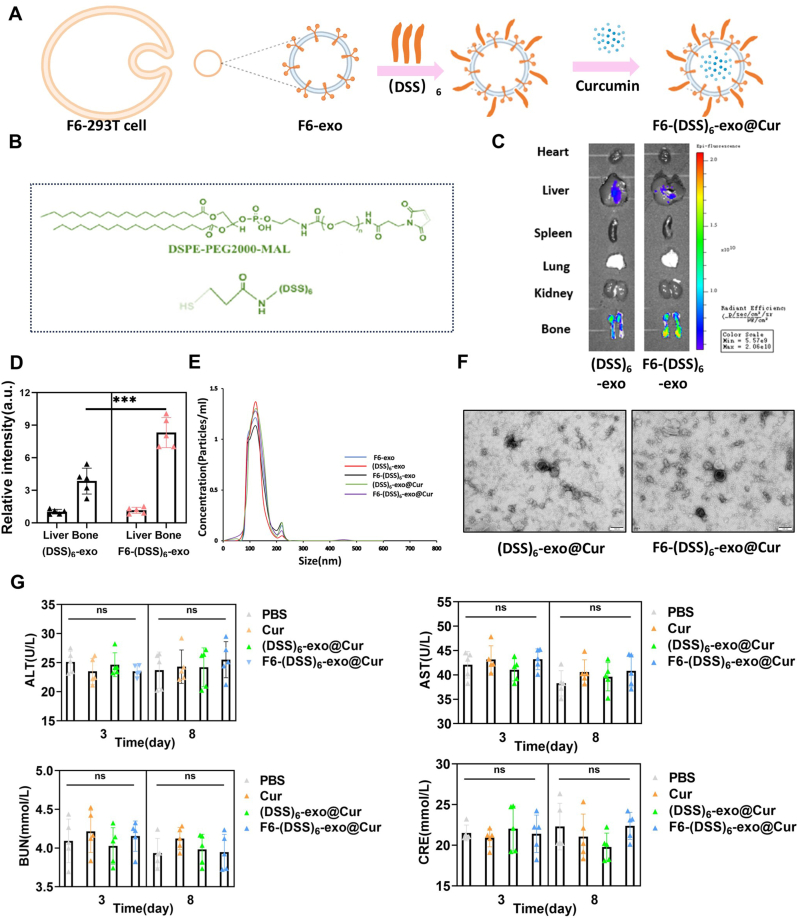
Preparation of bone-targeted nanoparticles and safety assessment in vivo. (A) Preparation of F6-(DSS)_6_-exo@Cur. (B) Synthetic routes of DSPE-PEG2000-(DSS)_6_. (C) Biophotonic imaging analysis revealed the organ distribution of Cy5-labeled nanoparticles 4 h after intravenous tail vein injection. (D) Relative fluorescence quantification of Cy5. (E) Diameter distribution of nanoparticles revealed by NTA. (F) Morphological images of (DSS)_6-_exo@Cur and F6-(DSS)_6-_exo@Cur revealed by transmission electron microscope. (G) After the injection of nanoparticles, the levels of ALT, AST, BUN, and CRE in the blood were measured. ∗∗∗P < 0.001. One-way ANOVA.

To address the intracellular accumulation of ferrous ions and ROS in BMSCs, we aim to use bone-targeting exosomes loaded with curcumin to inhibit ferroptosis in BMSCs. Traditional bone-targeting modifications typically involve inserting bone-targeting peptides on the surface of exosomes. Considering that the introduction of Fut6 is the result of genetic engineering, this represents a distinct modification approach. We hypothesize that the introduction of Fut6 could enhance the effect of conventional bone-targeting peptides. Therefore, we performed separate modifications of exosomes with bone-targeting peptides and dual modifications. As shown in Fig. 5A and B,two types of carriers, (DSS)_6_-exo and F6-(DSS)_6_-exo, were prepared and loaded with curcumin. To evaluate bone-targeting capability of the two carriers, Cy5-labeled nanoparticles were intravenously injected into mice, followed by biophotonic imaging analysis. Remarkably, F6-(DSS)_6_-exo exhibited enhanced bone-targeting efficiency compared to (DSS)_6_-exo (Fig. 5C and D). To verify the modification of (DSS)_6_, we labeled the bone-targeting peptide with the rhodamine fluorophore. Given the presence of CD63 on the surface of exosomes, we used immunomagnetic beads conjugated to an anti-CD63 antibody to capture the modified exosomes. The immunofluorescence results indicated that (DSS)_6_ was successfully anchored to the exosome surface (Figs. S3A–B). Similarly, we also successfully observed the loading of curcumin (Fig. S3C). After loading with curcumin, the particle sizes of both types of nanoparticles were comparable to those of regular extracellular vesicles (Fig. 5E). TEM revealed that both types of nanoparticles maintained the typical morphology of exosomes (Fig. 5F). Immunofluorescence of bone tissue confirmed that F6-(DSS)_6_-exo can deliver more curcumin to osteogenic cells (Figs. S4A and C). Compared to (DSS)6-exo, F6-(DSS)6-exo is more readily taken up by BEMCs (Figs. S4B and D). In addition, we measured the loading efficiencies of (DSS)_6_-exo@Cur and F6-(DSS)_6_-exo@Cur, which were 18.94 % and 18.77 %, respectively (Fig. S5A). The stability of both nanoparticles over 24 h was nearly identical (Fig. S5B). The biosafety and immunogenicity of F6-(DSS)_6_-exo@Cur were assessed to evaluate its potential for clinical applications. Hematological analysis demonstrated normal levels of alanine aminotransferase (ALT), aspartate aminotransferase (AST), blood urea nitrogen (BUN), and creatinine (CRE), indicating that F6-(DSS)_6_-exo@Cur treatment did not impair liver or kidney function (Fig. 5G).

### 3.5 F6-(DSS)_6_-exo@Cur treatment restores the osteogenic differentiation potential of BMSCs in SROP mice

**Fig. 6:**
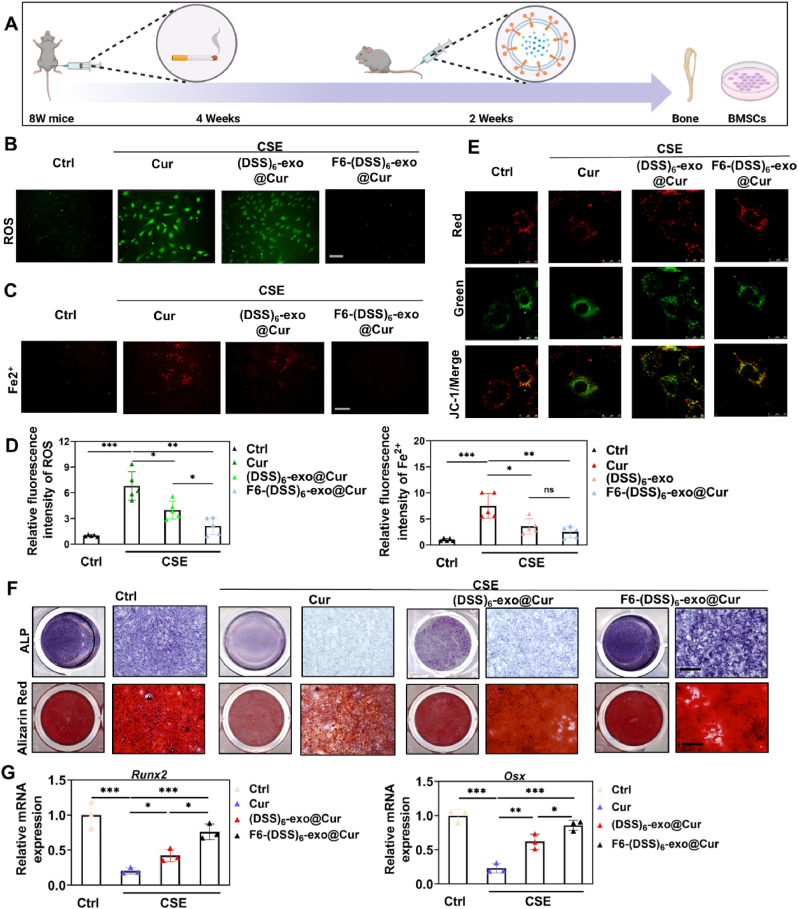
F6-(DSS)_6_-exo@Cur alleviated the accumulation of intracellular ROS and ferrous ions of BMSCs caused by CSE exposure and rescued their osteogenic differentiation potential in vivo.(A) Schematic diagram of SROP mice treated by nanoparticles.(B) Probe detection of intracellular ROS.(C) Probe detection of intracellular ferrous ions. (D) Relative fluorescence quantification of ROS and ferrous ions. (E) JC-1 staining of three groups of cells.(F)ALP and Alizarin Red staining. Scale bar: 400 μm(G)qPCR analysis of osteogenesis-related genes (Runx2, Osx) of BMSCs. ∗P < 0.05, ∗∗P < 0.01,∗∗∗P < 0.001. One-way ANOVA.

To evaluate the therapeutic efficacy of F6-(DSS)6-exo@Cur in vivo, SROP mice were treated with the nanoparticles (Fig. 6A). Post-treatment, BMSCs were isolated from the treated mice, and the results showed that non-targeted curcumin treatment had limited efficacy in managing SROP. Compared to (DSS)_6_-exo@Cur, F6-(DSS)_6_-exo@Cur significantly reduced ROS levels and ferrous ion accumulation in mouse BMSCs (Fig. 6B–D). Additionally, F6-(DSS)_6_-exo@Cur markedly restored the mitochondrial membrane potential in BMSCs, as evidenced by an increased red/green fluorescence ratio in JC-1 staining (Fig. 6E). Osteogenic differentiation was subsequently induced in BMSCs from treated SROP mice. Both alkaline phosphatase (ALP) and Alizarin Red staining demonstrated that F6-(DSS)_6_-exo@Cur treatment effectively restored the osteogenic differentiation potential of BMSCs (Fig. 6F). This was further confirmed by increased mRNA levels of osteogenic markers osterix (Osx) and runt-related transcription factor 2 (Runx2) (Fig. 6G). Finally, we assessed the impact of F6-(DSS)6-exo@Cur on key ferroptosis-related genes in CSE-exposed BMSCs. The results showed that F6-(DSS)6-exo@Cur downregulated ACSL4 and upregulated SLC7A11 and GPX4 (Fig. S6A). Additionally, F6-(DSS)6-exo@Cur significantly reduced MDA production (Fig. S6B).

### 3.6 F6-(DSS)_6_-exo@Cur treatment restores bone mass in SROP mice

**Fig. 7:**
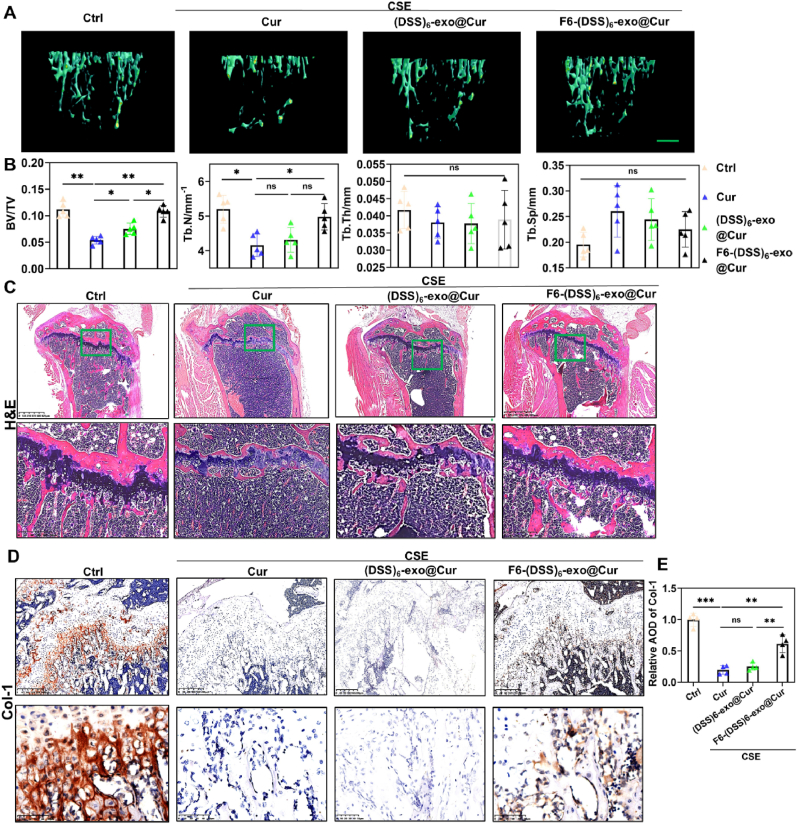
F6-(DSS)_6_-exo@Cur treatment rescued bone mass in SROP mice. (A) Microscopic CT three-dimensional reconstruction of proximal tibia. Scale bar: 200 μm. (B) Quantitative statistics of bone trabecular parameters (BV/TV, Tb. N, Tb. Sp, and Tb Th). (C) HE staining of proximal tibia. Scale bar: 625 μm and 300 μm. (D) Col-1 IHC staining of distal femur in control group and CSE exposed group. Scale bar: 200 μm and 50 μm. (E) The calculation of relative average optical density (AOD) of Col-1 (n = 4 in each group).∗P < 0.05, ∗∗P < 0.01,∗∗∗P < 0.001. One-way ANOVA.

Micro-CT analysis revealed that F6-(DSS)_6_-exo@Cur treatment significantly restored bone mass in SROP mice (Fig. 7A). Compared to the (DSS)_6_-exo@Cur group, BV/TV and Tb.N significantly increased in the F6-(DSS)_6_-exo@Cur group. However, no significant changes were observed in Tb. Sp orTb.Th (Fig. 7B). H&E staining demonstrated a marked increase in trabecular density in the proximal tibia of F6-(DSS)_6_-exo@Cur-treated mice (Fig. 7C). Furthermore, immunohistochemical staining showed elevated expression of Col-1 in the femurs of SROP mice treated with F6-(DSS)6-exo@Cur (Fig. 7D and E). These findings suggest that F6-(DSS)_6_-exo@Cur holds significant therapeutic potential for reversing osteoporosis in CSE-exposed mice.

## 4 Discussion

In this study, we successfully constructed a mouse model of SROP using cigarette smoke extract (CSE). Through this model, we investigated the specific mechanisms underlying CSE-induced osteoporosis. Transcriptome sequencing revealed that cigarette toxins impair the osteogenic differentiation potential of BMSCs by inducing ferroptosis, resulting in decreased bone mass. This finding highlights the potential of targeting ROS and ferrous ion accumulation in BMSCs as a therapeutic strategy. Curcumin, a widely studied ferroptosis inhibitor, has been shown to chelate metal ions such as ferrous and aluminum ions [41]. These chelation reactions reduce the metal load in vivo [42,43]. Additionally, curcumin can neutralize superoxide anion radicals generated by the xanthine oxidase system, as well as hydroxyl radicals produced during deoxyribose degradation and salicylic acid hydroxylation [44,45]. Curcumin has also been shown to alleviate oxidative stress by reducing lipid and protein oxidation caused by substances such as malondialdehyde and protein carbonyls [44]. Despite these properties, our results demonstrated that non-targeted curcumin injection exhibited limited therapeutic efficacy in treating SROP. This underscores the importance of developing targeted delivery systems, such as F6-(DSS)6-exo@Cur, to enhance the therapeutic potential of curcumin by specifically targeting bone tissue and mitigating ferroptosis in BMSCs.

As previously discussed, SROP is primarily mediated by the impaired osteogenic differentiation ability of BMSCs, necessitating cell-targeted therapeutic strategies. Exosomes are widely recognized for their utility as targeted delivery tools. Bone-targeting exosomes can be constructed by incorporating bone-targeting peptides through hydrophobic interactions. However, traditional co-incubation methods often result in limited peptide insertion and binding capacity, reducing therapeutic efficacy. Our findings highlight that peptide-modified exosomes, when constructed using traditional methods, exhibit suboptimal therapeutic performance. To address this limitation, we innovatively integrated genetic engineering to enhance the bone-targeting capabilities of peptide-modified exosomes. By leveraging the ligand-receptor binding principle, we targeted bone marrow endothelial cells expressing E-selectin by incorporating ESL into exosomes. Encouragingly, current research and our findings confirm that Fut6 substrates, such as CD44 and GLG1, exhibit a strong binding affinity to E-selectin [25,26]. Using Fut6 as an engineered gene, we successfully developed Fut6-exo, which demonstrated excellent bone marrow-targeting ability. The bone-targeted F6-(DSS)_6_-exo nanoparticle represents the most significant innovation of this research. Additionally, the extracellular vesicles used in this study can be produced in large quantities due to their derivation from cell lines, offering scalability for practical applications. Thus, our bone-targeted drug delivery system presents significant potential for treating orthopedic diseases. However, our study also has certain limitations. The related results are based on short-term experiments rather than long-term observations. The short-term efficacy of F6-(DSS)6-exo in drug delivery should not be taken as a representation of long-term outcomes. In-depth investigations into F6-(DSS)6-exo@Cur, such as long-term efficacy and off-target effects, will be one of the directions for our future research.

## 5 Conclusions

In summary, we identified Fut6 as a novel bone-targeting gene, representing a major innovation in bone-targeted therapy. By integrating genetic engineering with traditional bone-targeting peptide modifications, our approach demonstrates enhanced therapeutic efficacy and scalability. This research provides a promising and innovative bone-targeted delivery method for the treatment of SROP and other orthopedic diseases.

## CRediT authorship contribution statement

**Yao Wang:** Writing – original draft, Methodology, Investigation, Formal analysis, Data curation. **Lin Sun:** Software, Resources, Methodology, Data curation. **Zhenglin Dong:** Visualization, Software, Resources. **Tianyu Zhang:** Methodology. **Leining Wang:** Software. **Yihui Cao:** Resources. **Hui Xu:** Supervision, Funding acquisition. **Chenglei Liu:** Supervision, Funding acquisition. **Bo Chen:** Writing – review & editing, Supervision, Funding acquisition.

## Declaration of competing interest

The authors declare that they have no known competing financial interests or personal relationships that could have appeared to influence the work reported in this paper.

## Data Availability

Data will be made available on request.
