# Supplementary material for: Targeted inhibition of ferroptosis in bone marrow mesenchymal stem cells by engineered exosomes alleviates bone loss in smoking-related osteoporosis
Source: Mater Today Bio. 2025 Jan 21;31:101501. doi: 10.1016/j.mtbio.2025.101501 (PMC11815285; doi:10.1016/j.mtbio.2025.101501)
Supplement: Multimedia component 1 [file mmc1.docx]

**Supplementary material**

**Targeted inhibition of ferroptosis in bone marrow mesenchymal stem cells by engineered exosomes alleviates bone loss in smoking-related osteoporosis**

Yao Wang^a,d,1^,Lin Sun^d,1^ ,Zhenglin Dong^d,1^,Tianyu Zhang^c^,Leining Wang^a^,Yihui Cao^a^,Hui Xu^c,*^,Chenglei Liu^b,*^,Bo Chen^a,*^

**^a^** Department of Hand and Foot Surgery, The First Affiliated Hospital, Zhejiang University School of Medicine, Beilun,China

**^b^** Department of Radiology, Shanghai Ninth People's Hospital, Shanghai Jiao Tong University School of Medicine, Shanghai, China

**^c^** Bengbu first people’s hospital, Bengbu Medical University ,China

**^d^**Shanghai Key Laboratory of Orthopedic Implants, Department of Orthopedic Surgery, Shanghai Ninth People's

Hospital, Shanghai Jiao Tong University School of Medicine, Shanghai, China.

***Corresponding authors**

E-mail addresses: chenbo80@zju.edu.cn (B. Chen), lcl1984@aliyun.com( C. Liu), bbxuhui@hotmail.com (H. Xu)

^1^Yao Wang, Lin Sun and Zhenglin Dong contributed equally to this work.


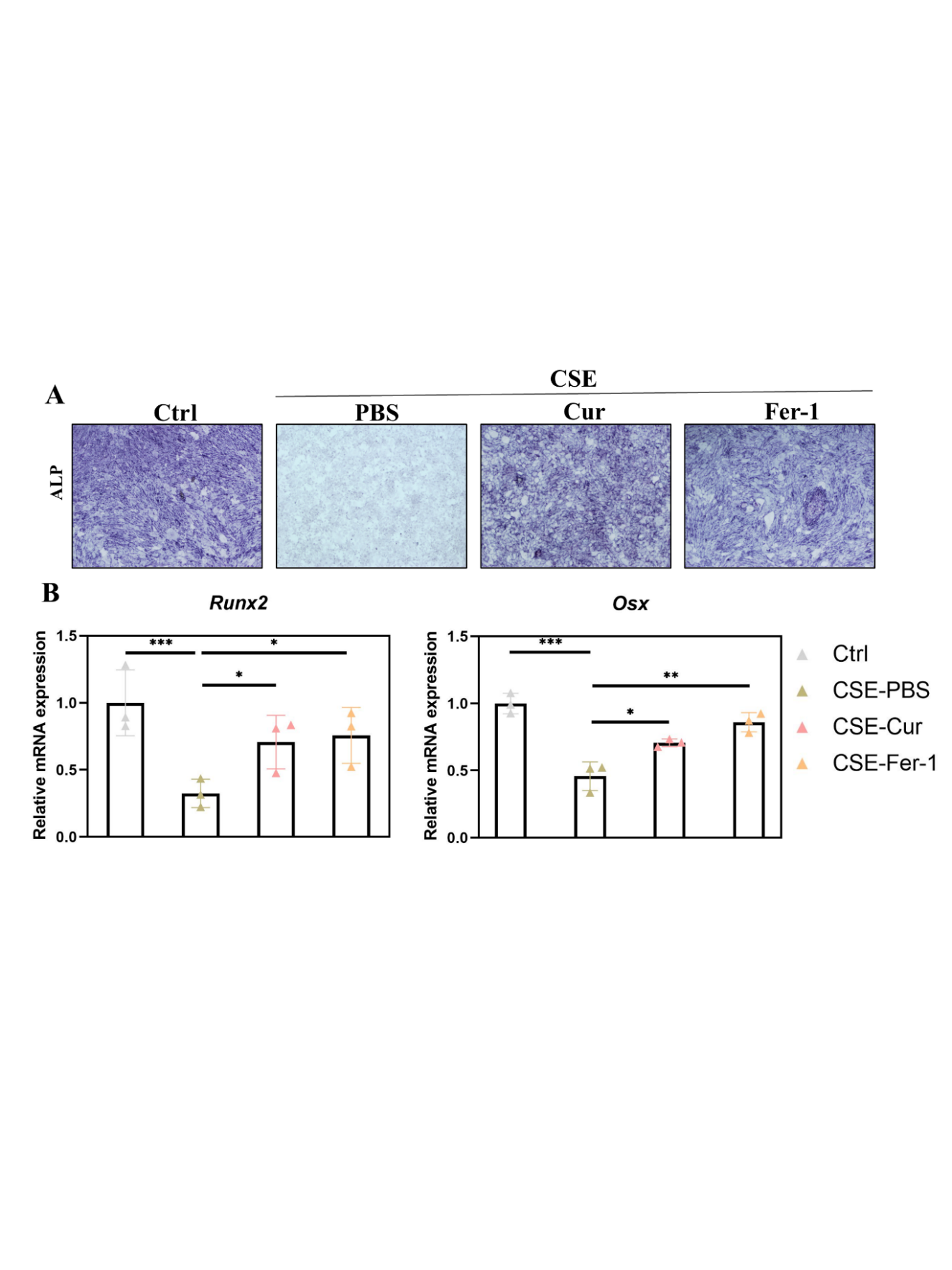


**Fig. S1. Inhibiting ferroptosis can rescue the osteogenic differentiation of CSE-exposed BMSCs.** (A) ALP staining of BMSCs after osteogenic induction. Scale bar: 200μm. (B) Rt-PCR analysis of RUNX2 and OSX mRNA levels. *p < 0.05, **p < 0.01. One-way ANOVA.

**
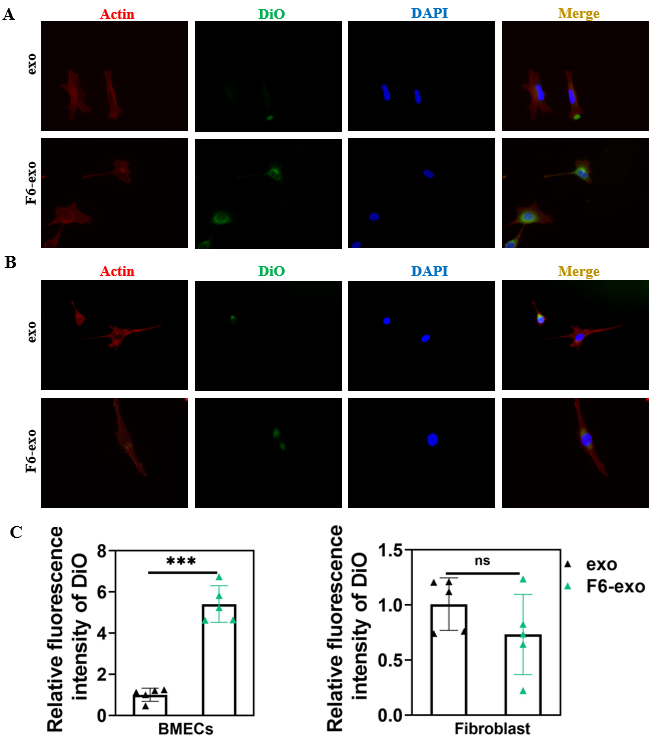
**

**Fig. S2.** **F6-exo preferentially binds to BMECs in the context of osteoporosis.** (A) The uptake of exosomes by bone marrow endothelial cells and lung fibroblasts (B). Scale bar: 50μm. (C) Statistical analysis of the fluorescence intensity of DiO. *** P<0.001. Student's t test.


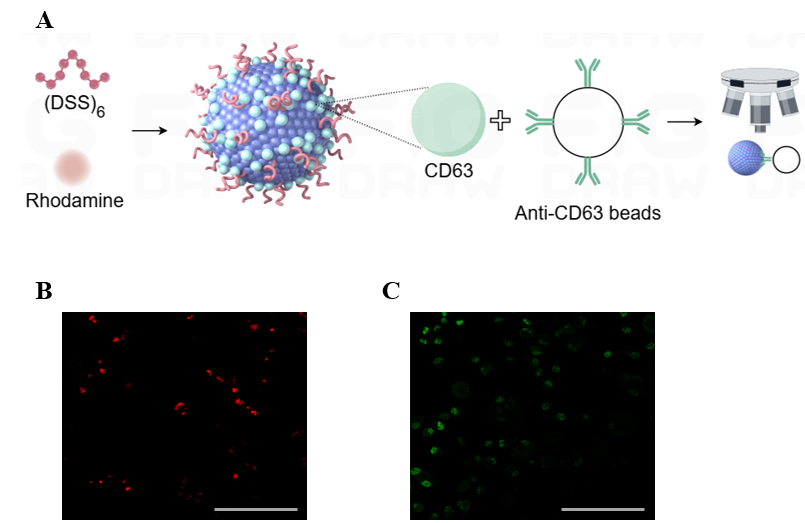


**Fig. S3. (DSS)_6_ and curcumin were successfully loaded.** (A) Schematic representation of (DSS)_6_ conjugated to a fluorophore and exosome capture. (B) Rhodamine fluorescence in exosomes. Scale bar:5μm. (C) Curcumin fluorescence in exosomes. Scale bar: 5 μm.

**
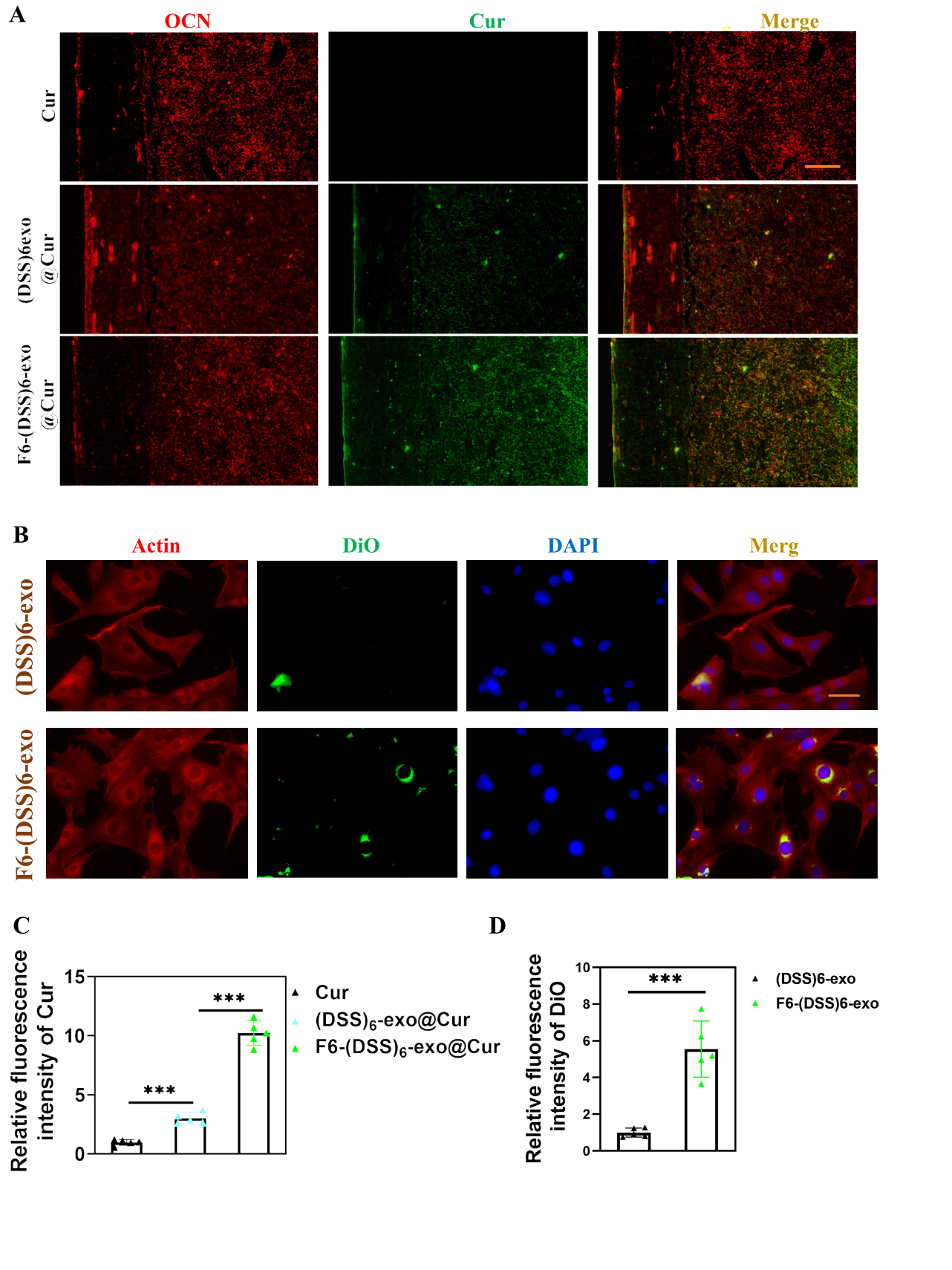
Fig. S4.** **F6-(DSS)6-exo exhibits enhanced bone targeting ability.** (A) F6-(DSS)6-exo delivered more curcumin to the bone marrow. Scale bar: 650μm. (B) F6-(DSS)6-exo preferentially binds to BMECs. (C) Statistical analysis of the fluorescence intensity of Cur. *** P <0.001. One-way ANOVA. (D) Statistical analysis of the fluorescence intensity of DiO. *** P <0.001. Student's t test.

For the loading efficiency, 50μL of exo@Cur was mixed with 1 mL of a 50/50 (v/v) acetonitrile/ethanol solution. The sample was then centrifuged to separate the exosomal proteins from the curcumin in the supernatant. The curcumin content in the supernatant was measured using high-performance liquid chromatography (HPLC). The protein pellets were dissolved in water, and the exogenous protein content was analyzed using the BCA method. The loading efficiency was calculated using the following formula:

amount of Cur in exo@Cur

Loading Efficiency (%) = ×100

amount of exosomal proteins in exo@Cur

In addition, the stability of curcumin in different carriers was assessed over 24 h in PBS at 37°C. Briefly, free curcumin and exo@Cur were added to PBS(PH=7.4), with a final concentration of 1.5μg/mL curcumin for each sample, and incubated in the dark at 37°C. At specified time (0 h, 4 h, 8 h, 16 h and 24 h), absorbance at 420 nm was measured for each sample using a TECAN spectrophotometer (TECAN, Männedorf, Switzerland). We took the fluorescence absorbance value of cur or exosomal cur at the beginning as the control group (100%). The data is obtained by dividing the values of the control group. As shown in the figure below, free curcumin degraded by more than 60% within the first 4 hours, with less than 20% remaining after 24 hours. In contrast, curcumin encapsulated in both exosomes retained more than 60% of its active component within 24 hours. Therefore, the stability of curcumin loaded in engineered exosomes significantly surpasses that of free curcumin.


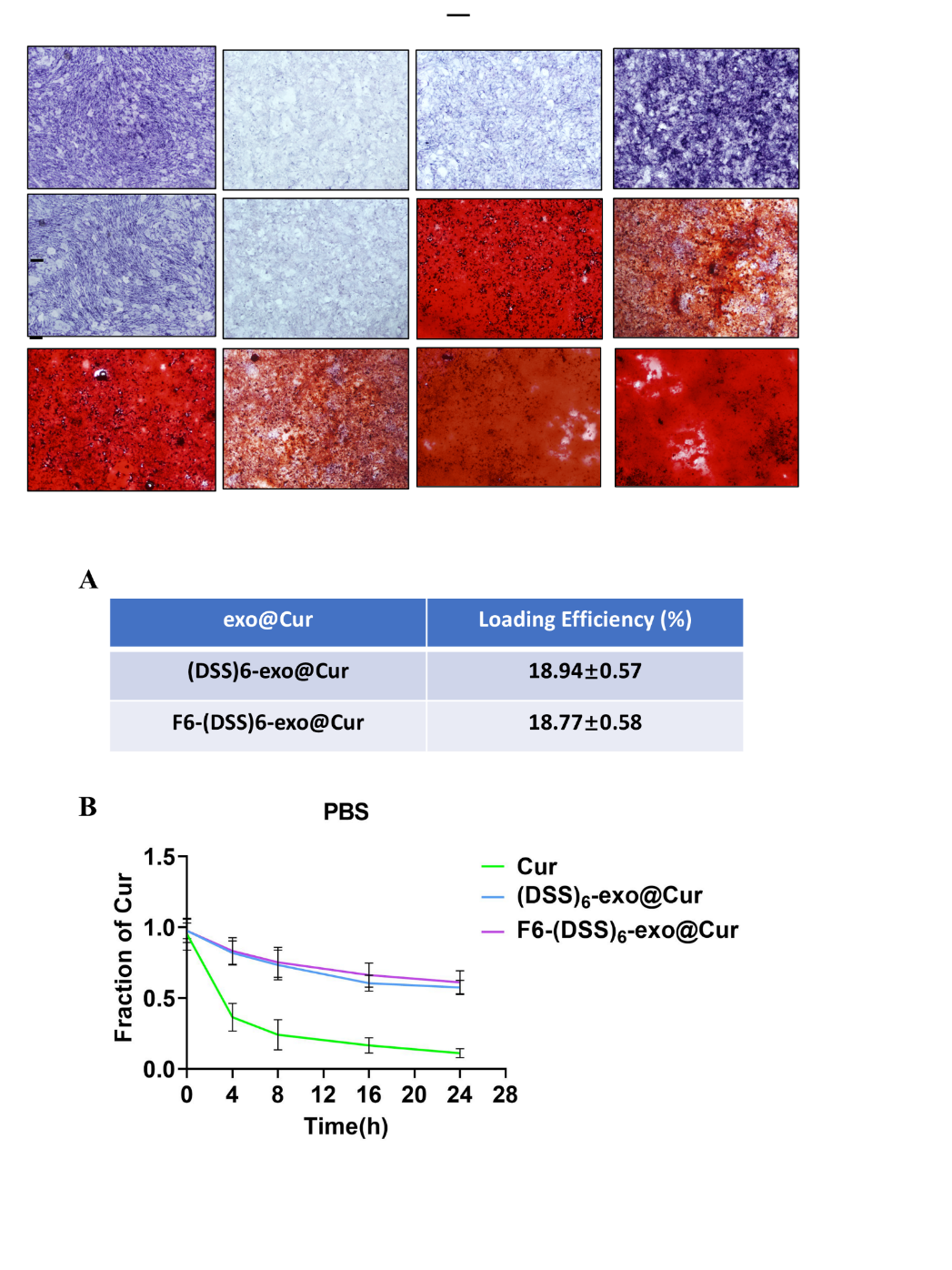


**Fig. S5. The loading efficiency and stability of exo@Cur.** (A) loading efficiency of two types of exo@Cur. (B) Stability analysis of free curcumin, (DSS)6-exo@Cur, and F6-(DSS)6-exo@Cur.


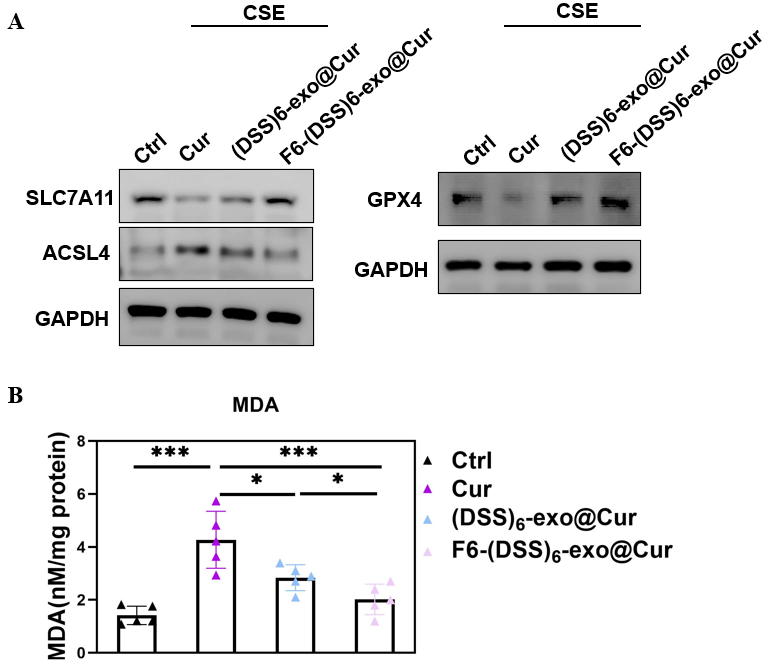


**Fig. S6.** **The F6-(DSS)6-exo@Cur regulates ferroptosis through the SLC7A11/GPX4 signaling axis.** (A-B) Western blotting was used to detect the expression levels of ACSL4,SLC7A11 and GPX4. (C)The content of malondialdehyde (MDA) was measured. * P<0.05, *** P<0.001. One-way ANOVA.
